# Supplementary figures and images for: Treacle’s ability to form liquid-like phase condensates is essential for nucleolar fibrillar center assembly, efficient rRNA transcription and processing, and rRNA gene repair
Source: eLife. 2025 Apr 14;13:RP96722. doi: 10.7554/eLife.96722 (PMC11996177; doi:10.7554/eLife.96722)

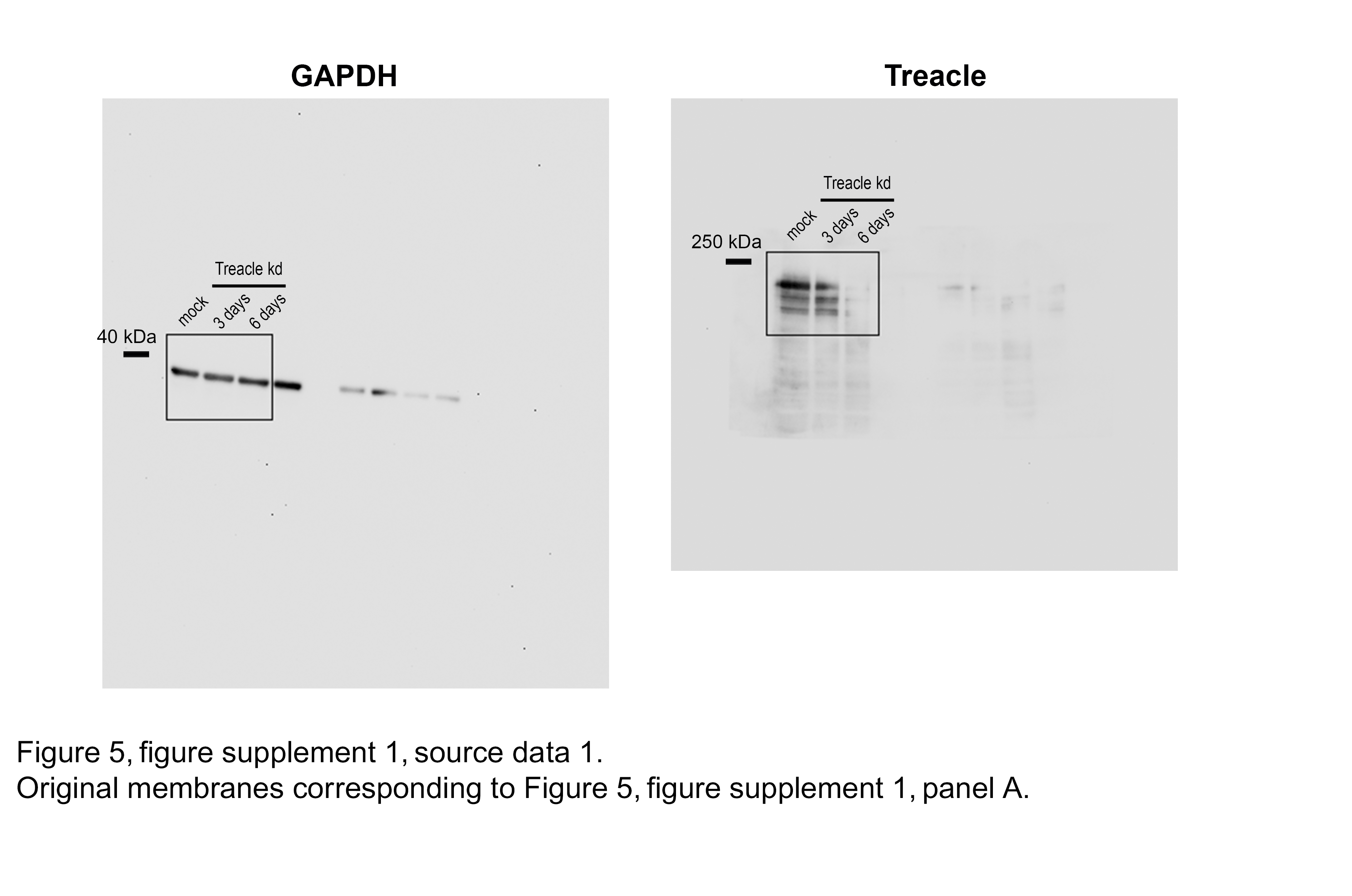

Supplement: Figure 5—figure supplement 1—source data 1. [file elife-96722-fig5-figsupp1-data1.zip › Figure 5–figure supplement 1—source data 1/Treacle_GAPDH.tif]

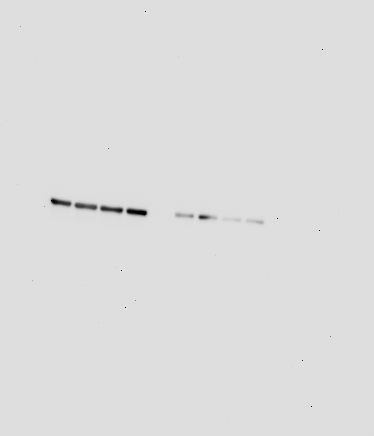

Supplement: Figure 5—figure supplement 1—source data 2. [file elife-96722-fig5-figsupp1-data2.zip › Figure 5–figure supplement 1—source data 2/GAPDH.tif]

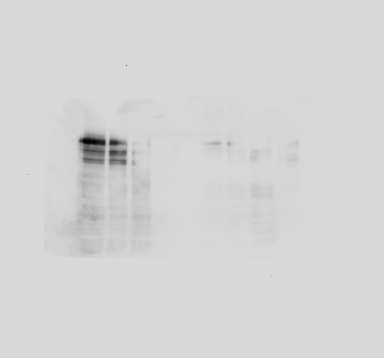

Supplement: Figure 5—figure supplement 1—source data 2. [file elife-96722-fig5-figsupp1-data2.zip › Figure 5–figure supplement 1—source data 2/Treacle.tif]

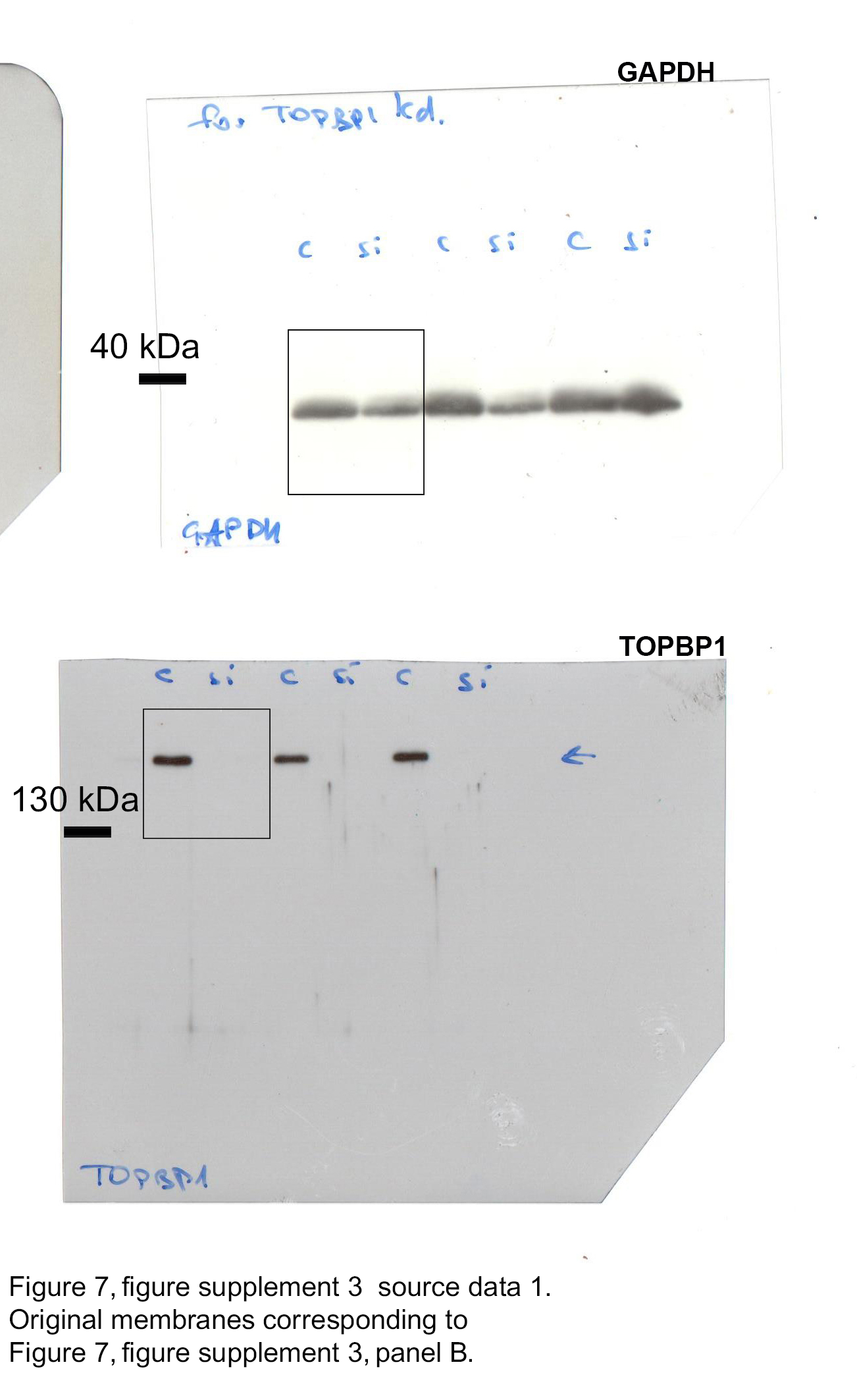

Supplement: Figure 7—figure supplement 3—source data 1. [file elife-96722-fig7-figsupp3-data1.zip › Figure 7–figure supplement 3 —source data 1/TOPBP1_GAPDH with labelled bands.tif]

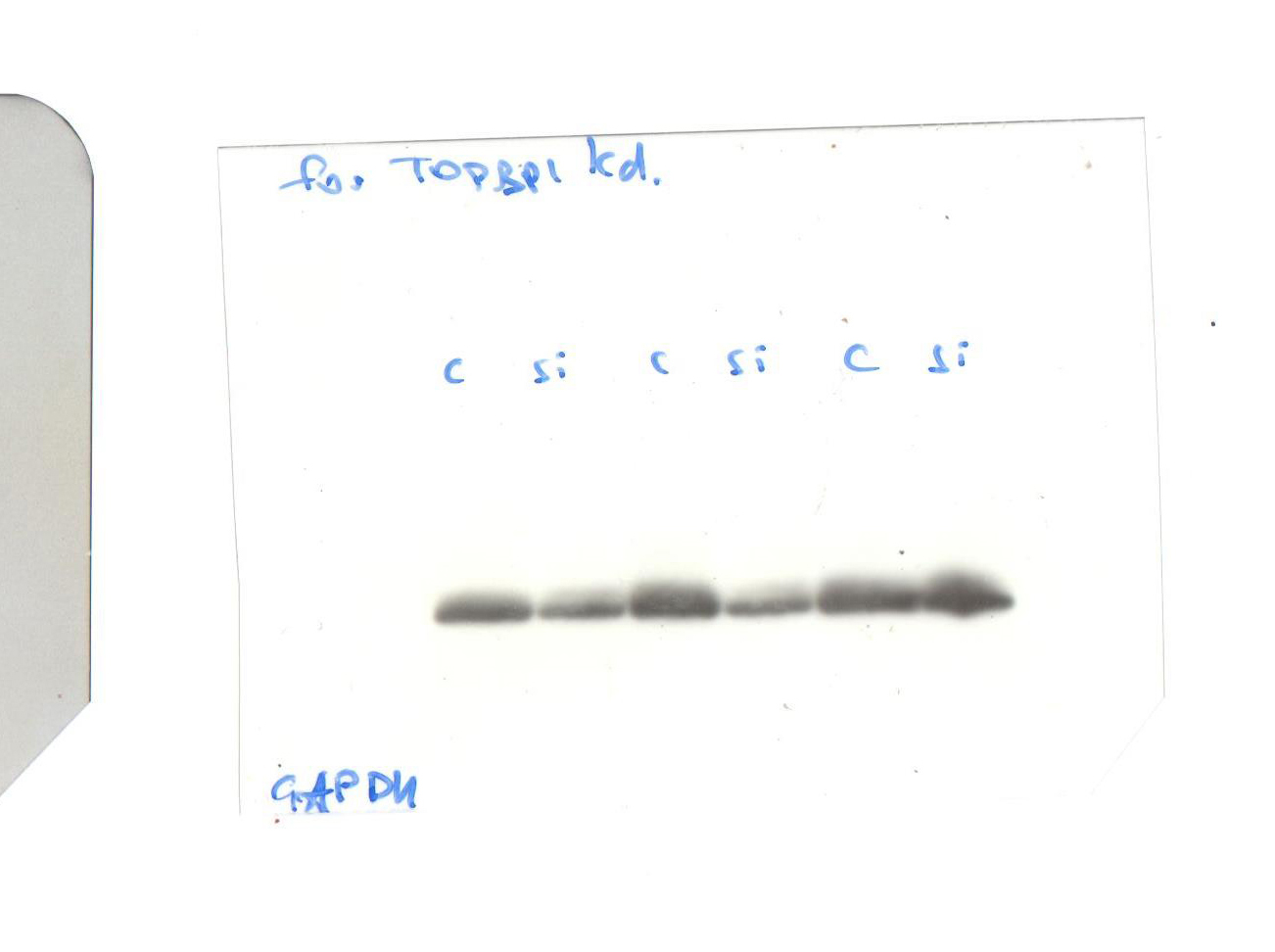

Supplement: Figure 7—figure supplement 3—source data 2. [file elife-96722-fig7-figsupp3-data2.zip › Figure 7–figure supplement 3 —source data 2/GAPDH.tif]

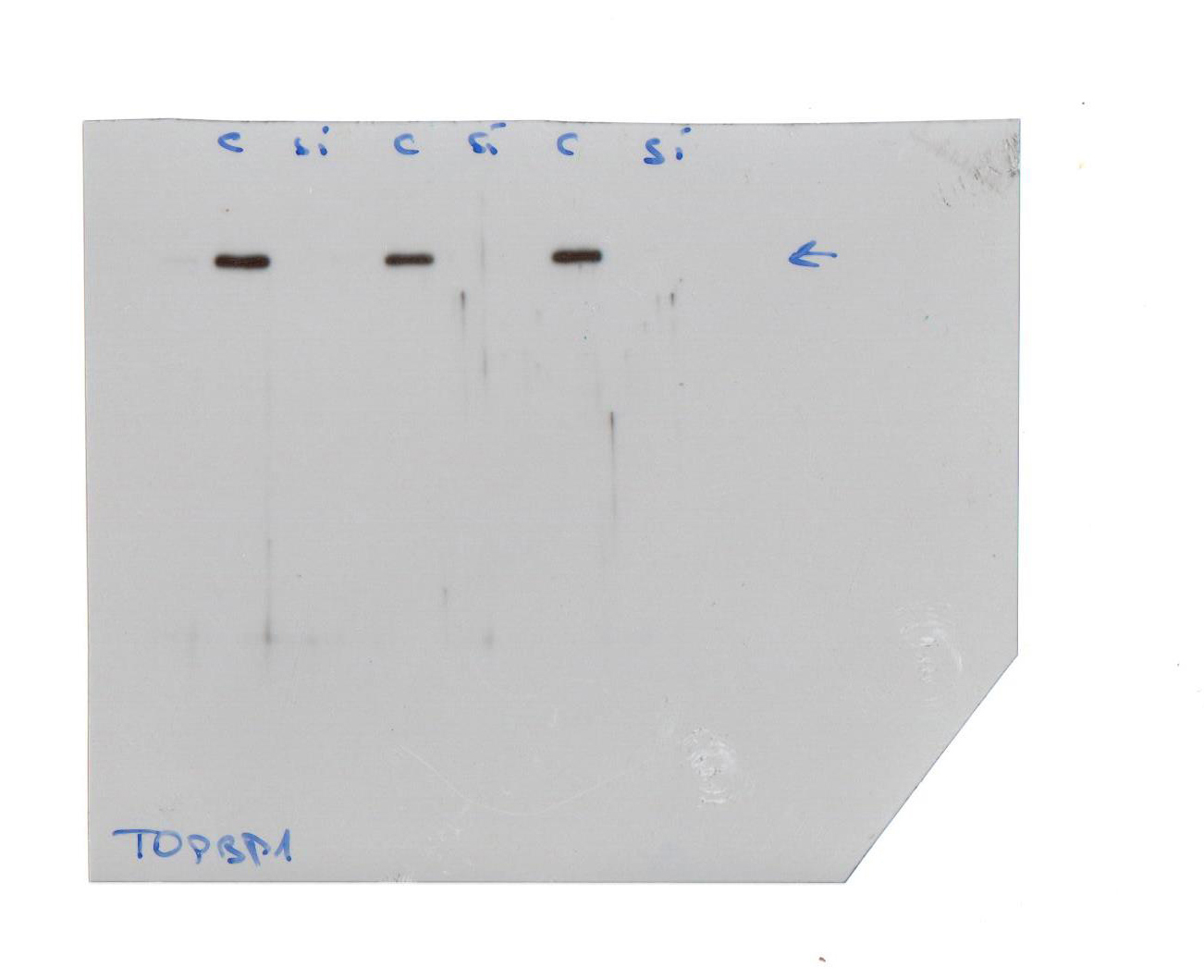

Supplement: Figure 7—figure supplement 3—source data 2. [file elife-96722-fig7-figsupp3-data2.zip › Figure 7–figure supplement 3 —source data 2/TOPBP1.tif]
