# Supplementary material for: Treacle’s ability to form liquid-like phase condensates is essential for nucleolar fibrillar center assembly, efficient rRNA transcription and processing, and rRNA gene repair
Source: eLife. 2025 Apr 14;13:RP96722. doi: 10.7554/eLife.96722 (PMC11996177; doi:10.7554/eLife.96722)
Supplement: Supplementary file 1. [file elife-96722-supp1.docx]

|  | List of primers used for cloning |  |
| --- | --- | --- |
|  | forward (5'-3') | reverse (5'-3') |
| Set#1 cloning | TAATCTCGAGGTATGGCCGAGGCCAGGAA | TAATCTCGAGTCATACAGTCTGCTCTGCTGTCTTC |
| Set#2 cloning | tatagctagcGCGTATGAAGATGGACAAAAAGACT | tatagctagcCGGGCTGCTGCTCCGAT |
| Set#3 cloning | TATATCCGGAGCTACCATGGCCTCAAACGATTATAC | TATAGGTACCCCTCCACGGTCCTGCTGTCCATAGC |
| Set#4 cloning | TATAAGATCTGTATGGCCGAGGCCAGGAA | TATAGGATCCTCATACAGTCTGCTCTGCTGTCTTC |
| Set#5 cloning | agcacctcggagagctcggaa | ggcagatctttcttcaccggcat |
| Set#6 cloning | tcagcccacacgctggtag | gatggggtctgacacacgg |
| Set#7 cloning | cccaagaagaagaggaaagtctaactgatcataatcagccataccacatttgtag | cttggccccctggggccctt |
| Set#8 cloning | TAATCTCGAGGTATGGCCGAGGCCAGGAA | TATAGGATCCAGCTGGCTGGTCTCCCGATAG |
| Set#9 cloning | TCCGGACTCAGATCTGCCAT | TAGCGGATCTGACGGTTCAC |
| Set#10 cloning | atatggatccgtaaaggcctctgaaaaaattctccaggtc | atatgtcgacttactgagcaggcgcctcctc |

| Added restrictive sites are highlighted in red |
| --- |
|  |
|  |
|  |
|  |
|  |
|  |
|  |
|  |
|  |
