# Supplementary material for: Treacle’s ability to form liquid-like phase condensates is essential for nucleolar fibrillar center assembly, efficient rRNA transcription and processing, and rRNA gene repair
Source: eLife. 2025 Apr 14;13:RP96722. doi: 10.7554/eLife.96722 (PMC11996177; doi:10.7554/eLife.96722)
Supplement: Supplementary file 2. [file elife-96722-supp2.docx]

Original Treacle full sequence

MAEARKRRELLPLIYHHLLRAGYVRAAREVKEQSGQKCFLAQPVTLLDIYTHWQQTSELGRKRKAEEDAALQAKKTRVSDPISTSESSEEEEEAEAETAKATPRLASTNSSVLGADLPSSMKEKAKAETEKAGKTGNSMPHPATGKTVANLLSGKSPRKSAEPSANTTLVSETEEEGSVPAFGAAAKPGMVSAGQADSSSEDTSSSSDETDVEGKPSVKPAQVKASSVSTKESPARKAAPAPGKVGDVTPQVKGGALPPAKRAKKPEEESESSEEGSESEEEAPAGTRSQVKASEKILQVRAASAPAKGTPGKGATPAPPGKAGAVASQTKAGKPEEDSESSSEESSDSEEETPAAKALLQAKASGKTSQVGAASAPAKESPRKGAAPAPPGKTGPAVAKAQAGKREEDSQSSSEESDSEEEAPAQAKPSGKAPQVRAASAPAKESPRKGAAPAPPRKTGPAAAQVQVGKQEEDSRSSSEESDSDREALAAMNAAQVKPLGKSPQVKPASTMGMGPLGKGAGPVPPGKVGPATPSAQVGKWEEDSESSSEESSDSSDGEVPTAVAPAQEKSLGNILQAKPTSSPAKGPPQKAGPVAVQVKAEKPMDNSESSEESSDSADSEEAPAAMTAAQAKPALKIPQTKACPKKTNTTASAKVAPVRVGTQAPRKAGTATSPAGSSPAVAGGTQRPAEDSSSSEESDSEEEKTGLAVTVGQAKSVGKGLQVKAASVPVKGSLGQGTAPVLPGKTGPTVTQVKAEKQEDSESSEEESDSEEAAASPAQVKTSVKKTQAKANPAAARAPSAKGTISAPGKVVTAAAQAKQRSPSKVKPPVRNPQNSTVLARGPASVPSVGKAVATAAQAQTGPEEDSGSSEEESDSEEEAETLAQVKPSGKTHQIRAALAPAKESPRKGAAPTPPGKTGPSAAQAGKQDDSGSSSEESDSDGEAPAAVTSAQVIKPPLIFVDPNRSPAGPAATPAQAQAASTPRKARASESTARSSSSESEDEDVIPATQCLTPGIRTNVVTMPTAHPRIAPKASMAGASSSKESSRISDGKKQEGPATQVSKKNPASLPLTQAALKVLAQKASEAQPPVARTQPSSGVDSAVGTLPATSPQSTSVQAKGTNKLRKPKLPEVQQATKAPESSDDSEDSSDSSSGSEEDGEGPQGAKSAHTLGPTPSRTETLVEETAAESSEDDVVAPSQSLLSGYMTPGLTPANSQASKATPKLDSSPSVSSTLAAKDDPDGKQEAKPQQAAGMLSPKTGGKEAASGTTPQKSRKPKKGAGNPQASTLALQSNITQCLLGQPWPLNEAQVQASVVKVLTELLEQERKKVVDTTKESSRKGWESRKRKLSGDQPAARTPRSKKKKKLGAGEGGEASVSPEKTSTTSKGKAKRDKASGDVKEKKGKGSLGSQGAKDEPEEELQKGMGTVEGGDQSNPKSKKEKKKSDKRKKDKEKKEKKKKAKKASTKDSESPSQKKKKKKKKTAEQTV

The replaced sequence is highlighted in yellow.

Insert Treacle ΔSE sequence

NSMPHPATGKTVANLLSGKSPRKSAEPSANTTLVSETEEEGSVPAFGAAAKPGMVSAGQADSSSEDTSSSSDETDVEGKPSVKPAQVKASSVSTKESPARKAAPAPGKVGDVTPQVKGGALPPAKRAKKPEEESESSEEGSESEEEAPAGTRSQVKASEKILQVRAASAPAKGTPGKGATPAPPGKAGAVASQTKAGKPEEDSESSSEESSDSEEETPAAKALLQAKASGKTSQVGAASAPAKESPRKGAAPAPPGKTGPAVAKAQAGKREEDSQSSSEESDSEEEAPAQAKPSGKAPQVRAASAPAKESPRKGAAPAPPRKTGPAAAQVQVGKQEEDSRSSSEESDSDREALAAMNAAQVKPLGKSPQVKPASTMGMGPLGKGAGPVPPGKVGPATPSAQVGKWEEDSESSSEESSDSSDGEVPTAVAPAQEKSLGNILQAKPTSSPAKGPPQKAGPVAVQVKAEKPMDNSESSEESSDSADSEEAPAAMTAAQAKPALKIPQTKACPKKTNTTASAKVAPVRVGTQAPRKAGTATSPAGSSPAVAGGTQRPAEDSSSSEESDSEEEKTGLAVTVGQAKSVGKGLQVKAASVPVKGSLGQGTAPVLPGKTGPTVTQVKAEKQEDSESSEEESDSEEAAASPAQVKTSVKKTQAKANPAAARAPSAKGTISAPGKVVTAAAQAKQRSPSKVKPPVRNPQNSTVLARGPASVPSVGKAVATAAQAQTGPEEDSGSSEEESDSEEEAETLAQVKPSGKTHQIRAALAPAKESPRKGAAPTPPGKTGPSAAQAGKQDDSGSSSEESDSDGEAPAAVTSAQVIKPPLIFVDPNRSPAGPAATPAQAQAASTPRKARASESTARSSSSESEDEDVIPATQCLTPGIRTNVVTMPTAHPRIAPKASMAGASSSKESSRISDGKKQEGPATQVSKKNPASLPLTQAALKVLAQKASEAQPPVARTQPSSGVDSAVGTLPATSPQSTSVQAKGTNKLRKPKL

The deleted SE residues

Insert Treacle CS sequence

NSMPHPATGETVANLLSGKSPEKSAEPSANTTLVSRTREEGSVPAFGAAAEPGMVSAGQADSSSRDTSSSSDRTDVEVEASEKILQVRAASAPAEGTPGKGATPAPPGEAGAVASQTKAGKPERDSESSSERSSDSRRETPAAKALLQAEASGKTSQVGAASAPAEESPRKGAAPAPPGETGPAVAKAQAGKRERDSQSSSRESDSRREAPAQAEPSGKAPQVRAASAPAEESPRKGAAPAPPRETGPAAAQVQVGKQERDSRSSSRESDSDREALAAMNAAQVEPLGKSPQVKPASTMGMGPLGKGAGPVPPGEVGPATPSAQVGKWERDSRSSSRESSDSSDGEVPTAVAPAQEESLGNILQAKPTSSPAEGPPQEAGPVAVQVKAEKPMDNSRSSRESSDSADSREAPAAMTAAQAEPALKIPQTEACPKKTNTTASAEVAPVRVGTQAPREAGTATSPAGSSPAVAGGTQRPAEDSSSSRESDSREEKTGLAVTVGQAESVGKGLQVKAASVPVEGSLGQGTAPVLPGETGPTVTQVKAEKQEDSRSSREESDSREAAASPAQVETSVKKTQAKANPAAAEAPSAKGTISAPGEVVTAAAQAKQRSPSKVEPPVRNPQNSTVLARGPASVPSVGEAVATAAQAQTGPERDSGSSREESDSRREAETLAQVEPSGKTHQIRAALAPAEESPRKGAAPTPPGETGPSAAQAGKQDDSGSSSRRSDSDGEAPAAVTSAQVIEPPLIFVDPNRSPAGPAATPAQAQAASTPREARASRSTARSSSSESRDEDVIPATQCLTPGIRTNVVTMPTAHPRIAPEASMAGASSSKESSRISDGKKQEGPATQVSEKNPASLPLTQAALKVLAQKASEAQPPVARTQPSSGVDSAVGTLPATSPQSTSVQAKGTNELRKPKL

The replaced E residues is highlighted in red, the replaced K residues is highlighted in blue.
