# Supplementary material for: Treacle’s ability to form liquid-like phase condensates is essential for nucleolar fibrillar center assembly, efficient rRNA transcription and processing, and rRNA gene repair
Source: eLife. 2025 Apr 14;13:RP96722. doi: 10.7554/eLife.96722 (PMC11996177; doi:10.7554/eLife.96722)
Supplement: Supplementary file 3. [file elife-96722-supp3.docx]

|  | **List of primers used for knockdown and knockout** |  |
| --- | --- | --- |
|  | forward (5'-3') | reverse (5'-3') |
| sgRNA1 TCOF | CACCGAAGTAGCTCCCGCCGCTTCC | AAACGGAAGCGGCGGGAGCTACTTC |
| sgRNA2 TCOF | CACCGGGGGTCGCGGGTATGGCCG | AAACCGGCCATACCCGCGACCCCC |
| siRNA TCOF | CCACCAUGGGUUGGAACUAAAСU-dTdT |  |

| Added restrictive sites are highlighted in red |
| --- |
|  |
