# Supplementary material for: Treacle’s ability to form liquid-like phase condensates is essential for nucleolar fibrillar center assembly, efficient rRNA transcription and processing, and rRNA gene repair
Source: eLife. 2025 Apr 14;13:RP96722. doi: 10.7554/eLife.96722 (PMC11996177; doi:10.7554/eLife.96722)
Supplement: Supplementary file 5. [file elife-96722-supp5.docx]

|  | **List of primers used for ChIP-qPCR** |  |
| --- | --- | --- |
|  | forward (5'-3') | reverse (5'-3') |
| ChIP d0 | GACAACGTGTATCTCTGCATT | ACACAGACACCTACATCTATCA |
| ChIP d1 | GGTATATCTTTCGCTCCGAGTC | ACAGGTCGCCAGAGGACAG |
| ChIP d3 | CAGCGTGTGCCTACCCTAC | TCCCTCGTTCATGGGGAATAA |
| ChIP d5 | TCCCTCCGAAGTTTCCCTCA | CGGCCCCAAGACCTCTAATC |
| ChIP d7 | CGCCTGGTCTTCTGTCTCTG | CGTAAGCTGGAGTGGAAGTGT |
