# Supplementary material for: Treacle’s ability to form liquid-like phase condensates is essential for nucleolar fibrillar center assembly, efficient rRNA transcription and processing, and rRNA gene repair
Source: eLife. 2025 Apr 14;13:RP96722. doi: 10.7554/eLife.96722 (PMC11996177; doi:10.7554/eLife.96722)
Supplement: Supplementary file 6. [file elife-96722-supp6.docx]

|  | **List FITS-labelled oligonucleotides used for smFISH** |
| --- | --- |
|  | (5'-3') |
| ETS-1-sm-1 rRNA FISH | AGAGGACAGCGTGTCAGC-FITS |
| ETS-1-sm-2 rRNA FISH | AACCTCTCCGACGACAGG -FITS |
| ETS-1-sm-3 rRNA FISH | CCGCGCGCATCCGGAGGC -FITS |
| ETS-1-sm-4 rRNA FISH | GTCACCGGTAGGCCAGAG -FITS |
| ETS-1-sm-5 rRNA FISH | GGAGCGCGGCCGGCTAGC -FITS |
| ETS-1-sm-6 rRNA FISH | CCCGGCAGGCGGCTCAAG -FITS |
| ETS-1-sm-7 rRNA FISH | GAGAGAACAGCAGGCCCG -FITS |
| ETS-1-sm-8 rRNA FISH | AGTCGGGACGCTCGGACG -FITS |
| ETS-1-sm-9 rRNA FISH | GGACCCGGGCCGGCACCG -FITS |
| ETS-1-sm-10 rRNA FISH | CCCGGGTGGGTCAGAGAC -FITS |
| ETS-1-sm-11 rRNA FISH | TCGCCCCCTTCCCCGCCG -FITS |
| ETS-1-sm-12  rRNA FISH | CGCACGGGGGCACGGTGG -FITS |
| ETS-1-sm-13 rRNA FISH | CGGGCGCCCGCAGCGGAG -FITS |
| ETS-1-sm-14 rRNA FISH | CGGGGTGGGGTTGTCGCG -FITS |
| ETS-1-sm-15 rRNA FISH | ACACGCACGGCACGGAGC -FITS |
| ETS-1-sm-16 rRNA FISH | CGCGGAGACGAGAACCCC -FITS |
| ETS-1-sm-17 rRNA FISH | GAAGGGGCGGCGGACAAC -FITS |
| ETS-1-sm-18 rRNA FISH | GGCCAACCCCCCACTCCG -FITS |
| ETS-1-sm-19 rRNA FISH | CCAGCGAGCCGATCGGCT -FITS |
| ETS-1-sm-20 rRNA FISH | AGCGGAGGCCGGCCGGCC -FITS |
